# Dynamic biological characteristics of human bone marrow hematopoietic stem cell senescence

**DOI:** 10.1038/s41598-022-21387-x

**Published:** 2022-10-12

**Authors:** Minhe Xiao, Peng Zhou, Ziling Wang, Hanxianzhi Xiao, Xiao Chen, Rong Jiang, Yaping Wang

**Affiliations:** 1 Department of Ophthalmology, The Third Affiliated Hospital of Chongqing Medical University, Chongqing, 401120 China; 2 Laboratory of Stem Cells and Tissue Engineering, Department of Histology and Embryology, Chongqing Medical University, Chongqing, 400016 China

**Keywords:** Senescence, Stem-cell research

## Abstract

Hematopoietic stem cells show biological manifestations of aging, diminished hematopoietic function and abnormal differentiation, which can lead to leukemia. It is therefore important to explore the mechanism underlying hematopoietic stem cell aging to develop strategies for delaying the process. Our evaluations revealed that the number of bone marrow hematopoietic cells (BMHCs) started to decrease significantly after 45 years of age, and the number of senescent BMHCs, as determined by senescence-associated beta-galactosidase staining, gradually increased with age. In addition, BMHCs from individuals over 45 years of age presented with notably reduced proliferative capacity, increased G1-phase cell cycle arrest, and significantly decreased generation of mixed colony forming units, which suggests that BMHCs enter senescence during middle age. Furthermore, we observed significantly lower antioxidant capacity and a significant increase in oxidative damage products, a gradual increase in the expression of senescence-associated proteins and genes, and a gradual decrease in the expression of cell cycle related proteins in BMHCs after middle age. Taken together, these findings offer both a theoretical and experimental basis for better understanding of the senescence progression of BMHCs and the optimal timing for anti-senescence drug interventions in clinical practice.

## Introduction

Both the blood and hematopoietic systems are essential to human life, with hematopoietic stem cells (HSCs) playing a critical role in maintaining the vitality of the hematological system. HSCs proliferate and differentiate into various hematopoietic progenitor cell lines and help maintain the dynamic equilibrium in blood cell counts and proportions. Organismal aging is accompanied by a gradual decline in the number and function of HSCs and previous studies^1,2^ suggest that there are age-related changes in HSCs, which manifest as increased susceptibility to anemia, compromised immunologic function and higher incidence of various diseases in elderly people. Moreover, hematopoiesis and the repopulation capacity of HSCs decline with age, thus lowering the proportion of HSCs differentiating into B lymphocytes and increasing the proportion of HSCs differentiating into granulocytes, resulting in corresponding changes in the gene expression profile of these progenitor cells^3–5^. These changes are closely associated with aging-induced decline in immunological functions^5^ and higher incidence of proliferative disorders in granulocytes in clinical practice^6^. Thus, it is essential to identify the underlying mechanisms of hematopoietic stem cell senescence and evaluate novel approaches for delaying senescence in these cells.

Our preliminary studies^7^ indicated that age-related changes in the HSCs of mice can cause a decline in their hematopoietic function, but further studies need to be conducted to validate these results and evaluate the senescence of human hematopoietic stem cells. This study collected bone marrow samples from healthy volunteers and divided the samples into four groups, based on age, to explore the temporal dynamics of the activity and proliferation of human HSCs, identify the potential mechanism of BMHC senescence in these populations, and provide experimental evidence and theoretical references for research into the mechanism of human HSC senescence and the development of anti-senescence drugs.

## Material and methods

### Ethics statement

All bone marrow specimens were obtained from outpatients screened in the hematology department at The First Affiliated Hospital of Chongqing Medical University, and the cohort comprised patients without any hematological diseases. The specimens were collected by bone marrow aspiration from the anterior superior iliac spine of the voluntary donors. The collected bone marrow specimens were treated with anticoagulants and divided into four groups by age: (1) < 30 years: 24 cases (13 females, 11 males); (2) 30–45 years: 24 cases (19 females, 5 males); (3) 46–60 years: 31 cases (10 females, 21 males); (4) > 60 years: 45 cases (13 females, 32 males).

### Reagents

StemSpan SFEM II, Serum-free Medium for Expansion of Hematopoietic Cells was sourced from STEMCELL Technologies (Seattle, WA, USA). The lymphocyte separation medium was sourced from Axis-Shield Diagnostics Ltd. (US). The red blood cell lysis buffer, BCA protein assay kit, senescence-associated beta-galactosidase (SA-β-gal) staining kit, cell lysis buffer, lipid peroxidation (MDA) assay kit, superoxide dismutase (SOD) assay kit, and reactive oxygen species (ROS) assay kit were sourced from Beyotime Biotech Co., Ltd. (China), while the EdU kit was sourced from RiboBio Co., Ltd. (China). The reverse transcription kit was sourced from TOYOBO biotech Co. (Japan) and the H4434 medium and complete hematopoietic cell culture medium were sourced from STEMCELL Technologies (US). The anti-P16, anti-P19, anti-P21 and anti-P53 monoclonal antibodies were sourced from Cell Signaling Technology (US).

### hBMHC isolation and culture

A total of 2–3 mL of bone marrow aspirate was collected from the anterior superior iliac spine of each voluntary donor under sterile conditions and placed in collection tubes containing a sodium heparin anticoagulant. The erythrocytes were lysed, and lymphocyte separation medium was used to isolate bone marrow mononuclear cells. After the cells were incubated with anti-CD34 antibody, they were sorted by magnetic field separator and washed with a special buffer for stem cells. The cells were quickly pushed out by the pistons matching the Mei Tian Biotec MS sorting column. The extracted cells were the human bone marrow CD34 + HSCs/HPCs required for the experiment. These cells were washed in phosphate buffered saline (PBS) and suspended in serum-free medium for expansion of hematopoietic cells before being subjected to short-period incubation in an incubator with saturated humidity and set at 5% CO_2_ and 37 °C. The culture bottles were checked once every 12 h and the non-adherent suspension cells were collected, centrifuged and washed before propagating them as pure hBMHC cultures in complete hematopoietic cell culture medium for later experiments.

### Bone marrow cell count

Briefly, 2 mL bone marrow sample from each volunteer was mixed with 4 mL normal saline to form a homogeneous cell suspension. Next, the suspension was centrifuged at 1000 rpm for 5 min and supernatant was discarded. The cells were re-suspended in 1 mL PBS and an aliquot was placed in a blood cell counting chamber to enumerate nucleated cells in the suspension.

### SA-β-gal staining

The SA-β-gal staining kit was sourced from Beyotime Biotech Co., Ltd. (China). The kit uses X-Gal as a substrate and generates a dark blue product catalyzed by senescence specific β-galactosidase. As a result, cells or tissues expressing beta-galactosidase turn blue and can be differentiated under a light microscope. Briefly, cells from different age groups were counted and adjusted to the same concentration before performing SA-β-gal staining experiments according to the manufacturer’s instructions. Cells with blue-stained nuclei were considered senescent cells. A wet plate was prepared for both observation and photography (Leica) and was used to observe the positively stained cells.

### Proliferation assay

We performed the CCK-8 and EdU assays to evaluate cell proliferation. We first adjusted the cell concentration (2.5 × 10^7^ cells/mL) for inoculation into 96-well plates and then treated each well with 20 μL of CCK-8 at days 0, 1, 2, 3, 4, 5, 6 and 7 post-inoculation and incubated them for 4 h before determining the change in absorbance at 450 nm using a microplate reader.

EdU is a thymine nucleoside analog, with associated alkynes that are rare among natural compounds. EdU can penetrate the DNA molecules in place of thymine (T) during DNA replication. Based on the specific reaction of Apollo® fluorescent dye and EdU, DNA replication can be detected directly and accurately. For the EdU assay, cells from each group were plated (1.5 × 10^5^ cells/mL/well) onto 24-well plates and processed as per the manufacturer’s instructions before being imaged with a fluorescence microscope. ImageJ software was used to calculate the number of EdU-Hoechst 33,342-positive cells in each sample.

### Cell cycle analysis

The bone marrow of each group was sorted, purified and cultured in presence of hematopoietic stem cell medium for 24 h. The cell density was adjusted to 1 × 10^6^ cells/mL/tube and the cells were fixed with 70% ethanol for 12 h. After centrifugation, the supernatant was discarded, and the cells were incubated with 100 μL pancreatic ribonuclease at 37 °C for 30 min. Then, cells were stained with propidium iodide, evaluated on a FACSCalibur (BD, Biosciences, Heidelberg, Germany), and analyzed using MoD fit LT for NAC V3.0.

### Detection of oxidation-associated biomarkers

Cells from each group were washed with PBS, lysed in an ice bath for 30 min, and centrifuged (12,000 rpm, 4 ℃, 30 min) to collect the supernatant. Activities of glutathione (GSH), reactive oxygen species (ROS), and Superoxide dismutase (SOD), and malondialdehyde (MDA) concentration were detected by chemical colorimetric analyses using various commercially available kits per the manufacturer’s instruction (Beyotime Institute of Biotechnology, Shanghai, China).

### In vitro production of colony forming unit (CFU)-mix

Cells (5 × 10^4^ cells/well) were plated in a 24-well plate and mixed with 1 mL semi-solid medium (#H4334, MethoCult™ H4434 Classic, STEMCELL Technologies, US) and hematopoietic progenitor cells (3 replicates/group). The remaining space in the wells was filled with PBS to prevent dehydration and the cells were incubated at 37 ℃ for 15 days, the CFU-mix comprised a mixed colony of granulocytes, red blood cells, macrophages, and megakaryocytes formed^8^. The number of CFU-Mix per sample were observed and enumerated using an inverted phase contrast microscope (OLYMPUS microscope, Japan) and ImageJ software.

### RNA extraction and RT-PCR

Total RNA was extracted from each of the hBMHC samples and reverse-transcribed using kits per manufacturer’s instructions (TaKaRa, Japan). SYBR green was used for fluorescence-based quantitative PCR and each sample was evaluated in triplicate. The primers used in each reaction are listed in Table 1. β-actin was used as the reference gene. Each reaction mixture (10 μL) included SYBR® Premix Taq™II (2 ×) 5 μL, forward primer (10 μΜ) 0.1 μL, reverse primer (10 μΜ) 0.1 μL, cDNA, 2 μL, RNase-Free dH_2_O 2.8 μL, and the reaction conditions were as follows: 40 cycles of 30 s at 95 ℃, 5 s at 95 ℃ and 30 s at 60 ℃. The ΔCq value was used to determine the relative expression level of each target gene, where ΔCq = Cq (target gene)—Cq (internal reference gene).

**Table 1:** Primers used in Real-time quantitative PCR.

p53	Forward	5′-ACCGGCGCACAGAGGAAGAG-3′
	Reverse	5′-CTGGGGAGAGGAGCTGGTGTTG-3′
p21	Forward	5′-AGGCCCGTGAGCGATGGAAC-3′
	Reverse	5′-CGCCTGCCTCCTCCCAACTC-3′
p19	Forward	5′-TTCGCGCCTGGCATTACATC-3′
	Reverse	5′-GTGGGGGAAGGCATATATCTACG-3′
p16	Forward	5′-GGCACCAGAGGCAGTAACCATG-3′
	Reverse	5′-GCCAGCCAGCTTGCGATAAC-3′
β-actin	Forward	5′-ACCCCGTGCTGCTGACCGAG-3′
	Reverse	5′-TCCCGGCCAGCCAGGTCCA-3′

### Western blot analysis

Total protein was extracted from the hematopoietic cells obtained from each group and quantified using the BCA protein assay kit. The concentrations of the protein lysates were adjusted (40 µg/well) before they were analyzed by SDS-PAGE. The resolved protein bands were electroblotted onto a PVDF membrane and their molecular weights were determined using the Pageruler™ Prestained Protein Ladder (Thermo Scientific, #26616). The membrane was then blocked with 5% skim milk for 2 h at room temperature. The blots were then cut and incubated with the relevant monoclonal primary antibodies (1:2000) overnight at 4 ℃. Next, the PVDF membrane was washed with Tris buffered saline Tween-20 (TBST) and incubated with HRP-conjugated goat anti-rabbit or rabbit anti-mouse secondary antibodies (1:5000) for 2 h at room temperature before being exposed to ECL reagent, imaged and analyzed using Image Lab 5.2.1 software. The bands were quantified based on their relative intensity.

### Statistical analysis

SPSS 18.0 software was used for data analysis. One-way analysis of variance was performed to analyze the data obtained from each experiment. Differences were considered statistically significant at *p* < 0.05 and the data are represented as the mean value ± standard deviation (x ± s) of the experimental triplicates.

### Ethics approval and consent to participate

Bone marrow collection followed the WMA Declaration of Helsinki. Our study protocol was approved by the Ethics Committee of the First Affiliated Hospital of Chongqing Medical University and the bone marrow specimens were collected following the receipt of written informed consent from each of the registered donors.

## Results

### hBMHCs isolated from aged volunteers showed increased senescence

**Figure 1:**
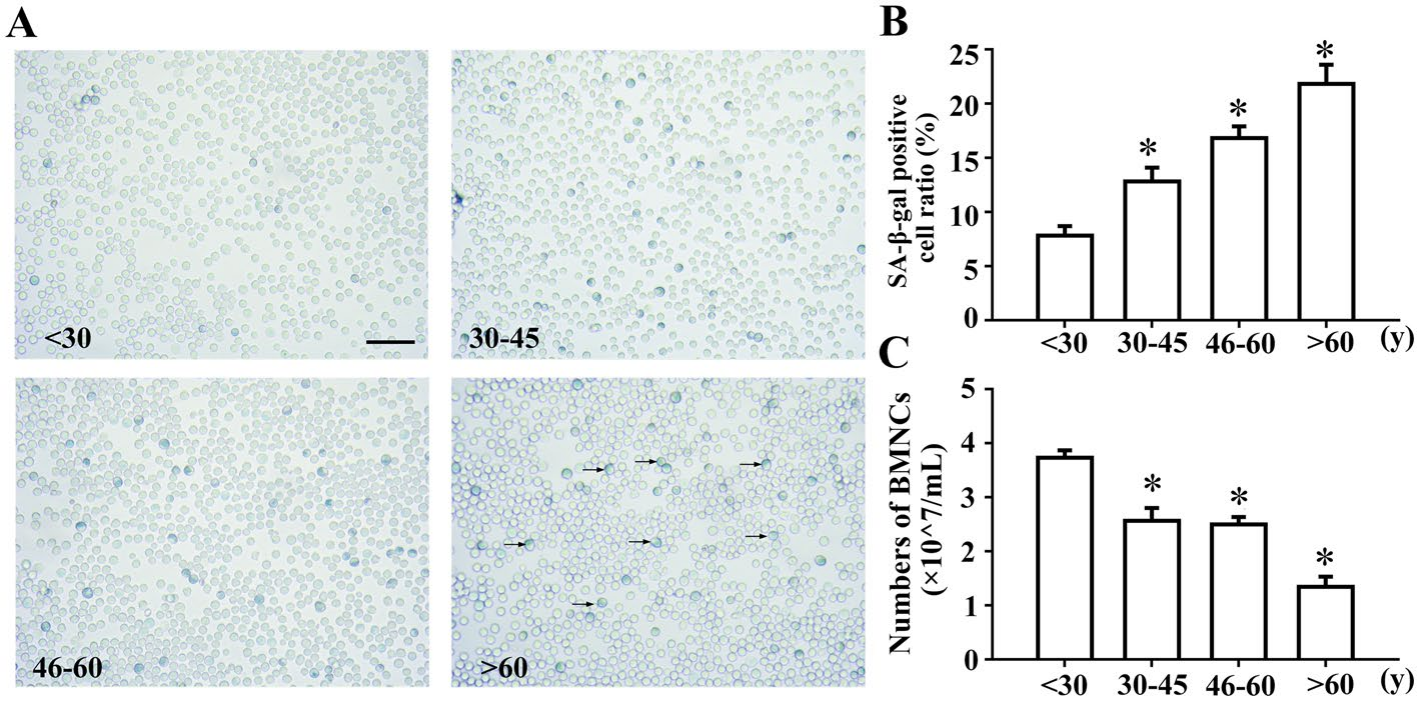
Aging of hBMHCs in different age groups. (**A**) Representative image of the SA-β-gal staining. (**B**) Graphical representation of the enumeration of SA-β-gal positive cells in each group. (**C**) hBMHC count in different age groups (× 10^7^/mL), *p < 0.05: compared with that in the < 30 years group. Bar = 50 μm. “ → ”: positive cells.

Human bone marrow nucleated cell counts and SA-β-gal staining were used to evaluate the senescence of human bone marrow cells. The number of nucleated cells that could be isolated from an equal number of bone marrow samples gradually decreased with age, and the results showed a significant decrease in the total number of cells in the > 60 years age group compared with that in the < 30 years age group (Fig. 1C). SA-β-gal staining of CD34 + magnetically separated progenitor cells showed that the number of SA-β-gal-positive cells increased with age, indicating increased senescence in these samples (Fig. 1A,B).

### Decreased proliferative capacity and increased cell cycle arrest of hBMHCs with age

**Figure 2:**
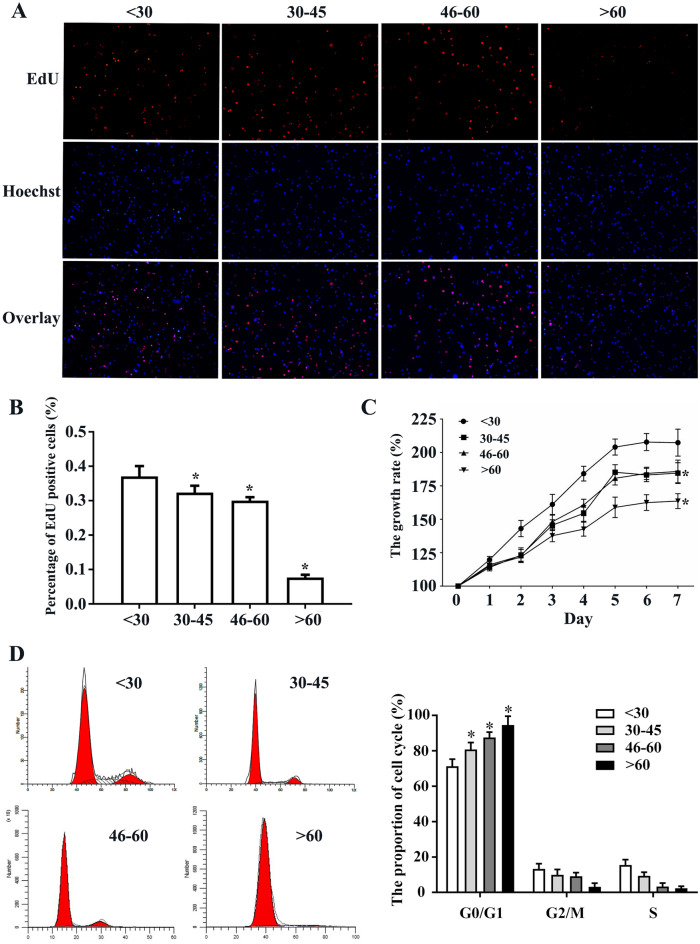
Cell cycle analysis and proliferation rates of hBMHCs obtained from individuals of different age groups. (**A**) Representative image of the EdU staining. (**B**) Graphical representation of the enumeration of EdU positive cells in each group. (**C**) CCK-8 assay was performed to determine growth rates in cells obtained from individuals of different ages. (**D**) Representative image of the cell-cycle and graphical representation of the enumeration of cell-cycle in each group. *p < 0.05: when compared with the < 30 years group. Scale bar = 200 µm.

Cellproliferation was determined using total monocytes after removal of adherent cells. The CCK-8 results suggest that the proliferative capacity of hBMHCs decreases with age (Fig. 2C). The proliferative capacity of the hematopoietic cells from each group gradually plateaued after 7 days. EdU results also suggested that the proliferative capacity of the hBMHCs gradually decreased with age (Fig. 2A,B). Total monocytes after removal of adherent cells were used for flow cytometry analyses and the results showed that the hBMHCs experienced increasing G1-phase cell cycle arrest as they aged (Fig. 2D), indicating that cell cycle arrest is likely correlated with senescence in these samples.

### Self-renewal and differentiation capacity of CFU-Mix decreased with age

**Figure 3:**
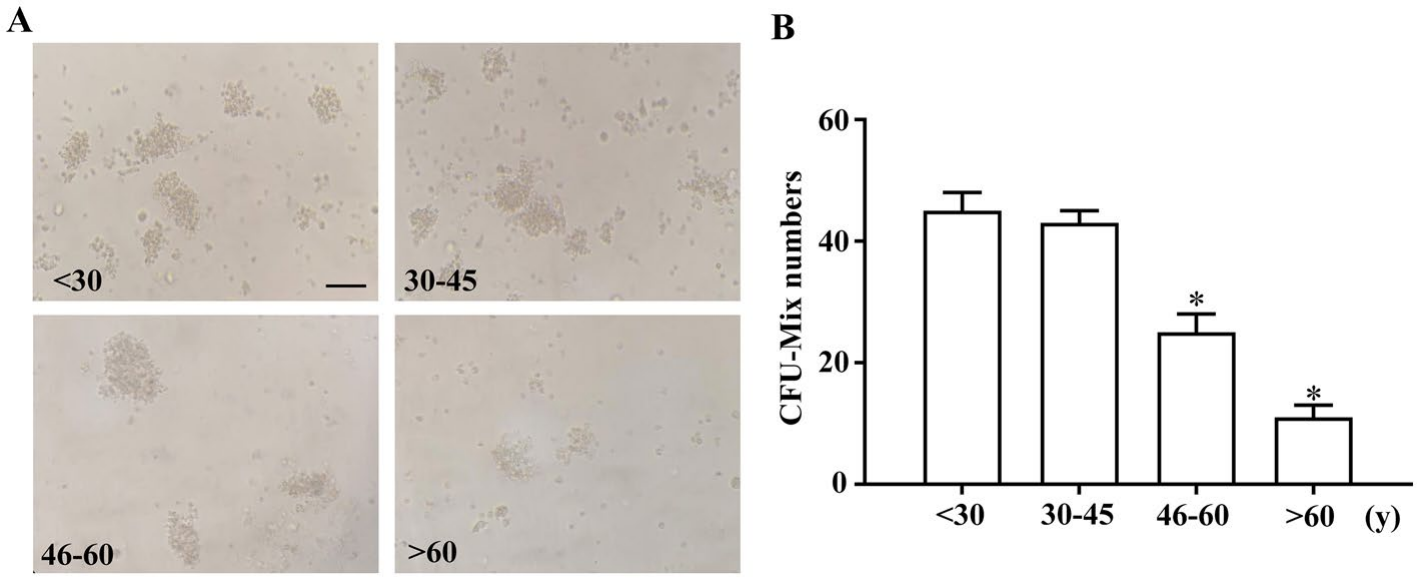
Comparison of the amount of CFU-Mix formed by hBMHCs obtained from different age groups. (**A**) Representative image of the CFU-Mix. (**B**) Graphical representation of the enumeration of CFU-Mix in each sample group. *p < 0.05: when compared with the < 30 years group. Scale bar = 100 µm.

HSCs formed colonies containing granulocytes, erythrocytes, macrophages and megakaryocytes after 12–16 days of culture. The formation of CFU-Mix reflects the ability of HSCs to self-renew and differentiate. We observed that the number of CFU-Mix colonies formed by an equal number of CD34 + magnetically selected progenitor cells decreased with age (Fig. 3A), with both the 46–60 and > 60 years age groups demonstrating a significant reduced CFU-Mix numbers compared to those observed in the > 30 years age group (Fig. 3B).

### hBMHCs of aged individuals showed oxidation/antioxidant system imbalance and increased mRNA expression of p53 and p21 Cip1/Waf1

**Figure 4:**
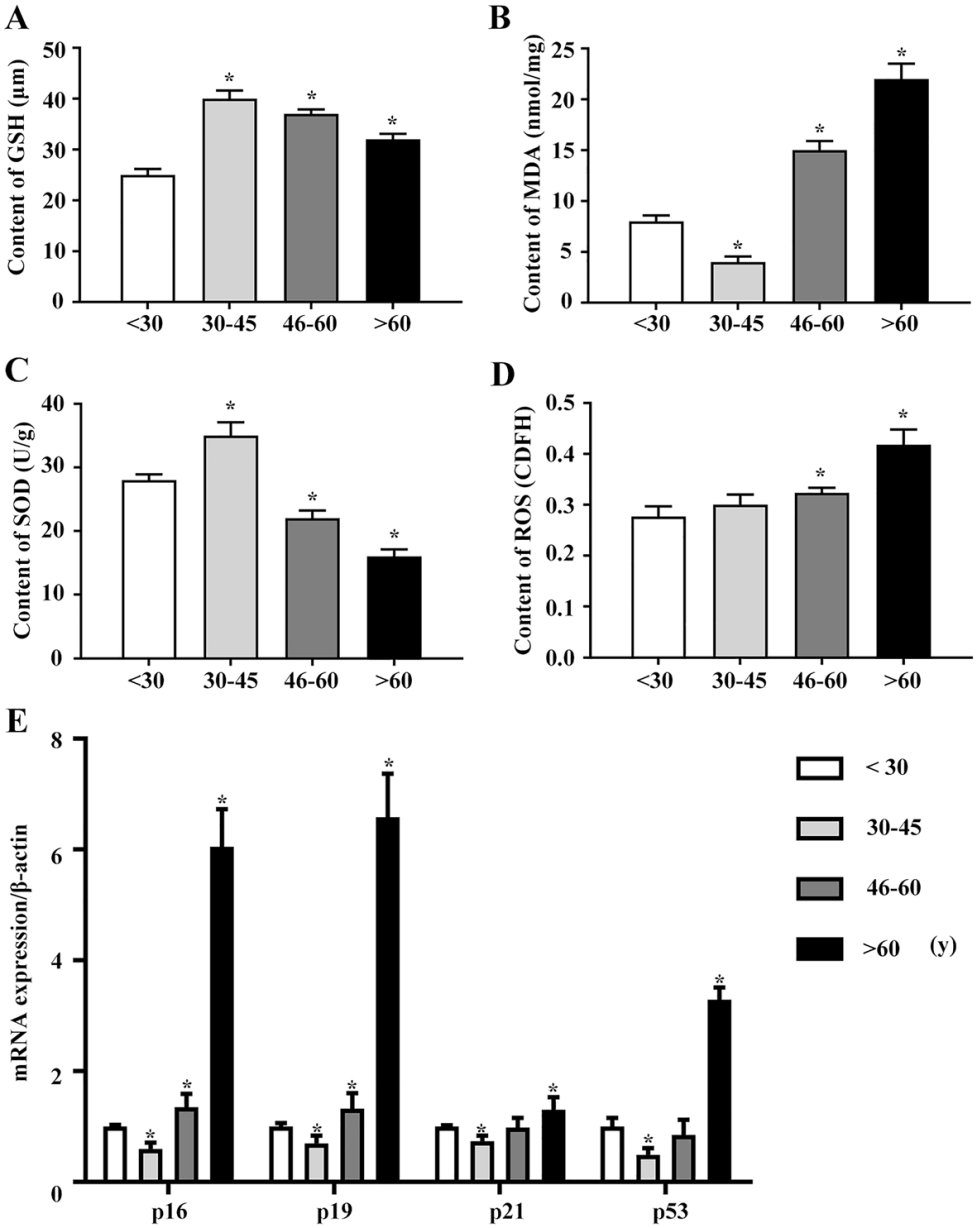
Oxidation/antioxidant system and mRNA expression of p53 and p21 Cip1/Waf1 in hBMHCs obtained from the individuals of different age groups. (**A**) Graphical representation of the GSH content in each sample group. (**B**) Graphical representation of the MDA content in each sample group. (**C**) Graphical representation of the SOD activity in each sample group. (**D**) Graphical representation of the ROS level in each sample group. *p < 0.05: when compared with the < 30 years group. (**E**) mRNA expression of p16, p19, p21, and p53 in hBMHCs obtained from different age groups. *p < 0.05: when compared with the < 30 years group.

The total monocyte lysates after the removal of adherent cells were used to measure the level of oxidative damage. The hBMHCs obtained from aged volunteers showed increased malondialdehyde (MDA) content and ROS (Fig. 4B,D), decreased SOD and TGSH-PX activities (Fig. 4A,C). The p53-p21 pathway is an important regulator of senescence. qRT-PCR analysis showed significantly increased expression of p53 and p21 in hBMHCs obtained from the older age groups (Fig. 4E).

### hBMHCs from aged individuals showed differential expression of P16, P19, P21, P53, CDK2, CDK4, CyclinD1 and CyclinE1 proteins

**Figure 5:**
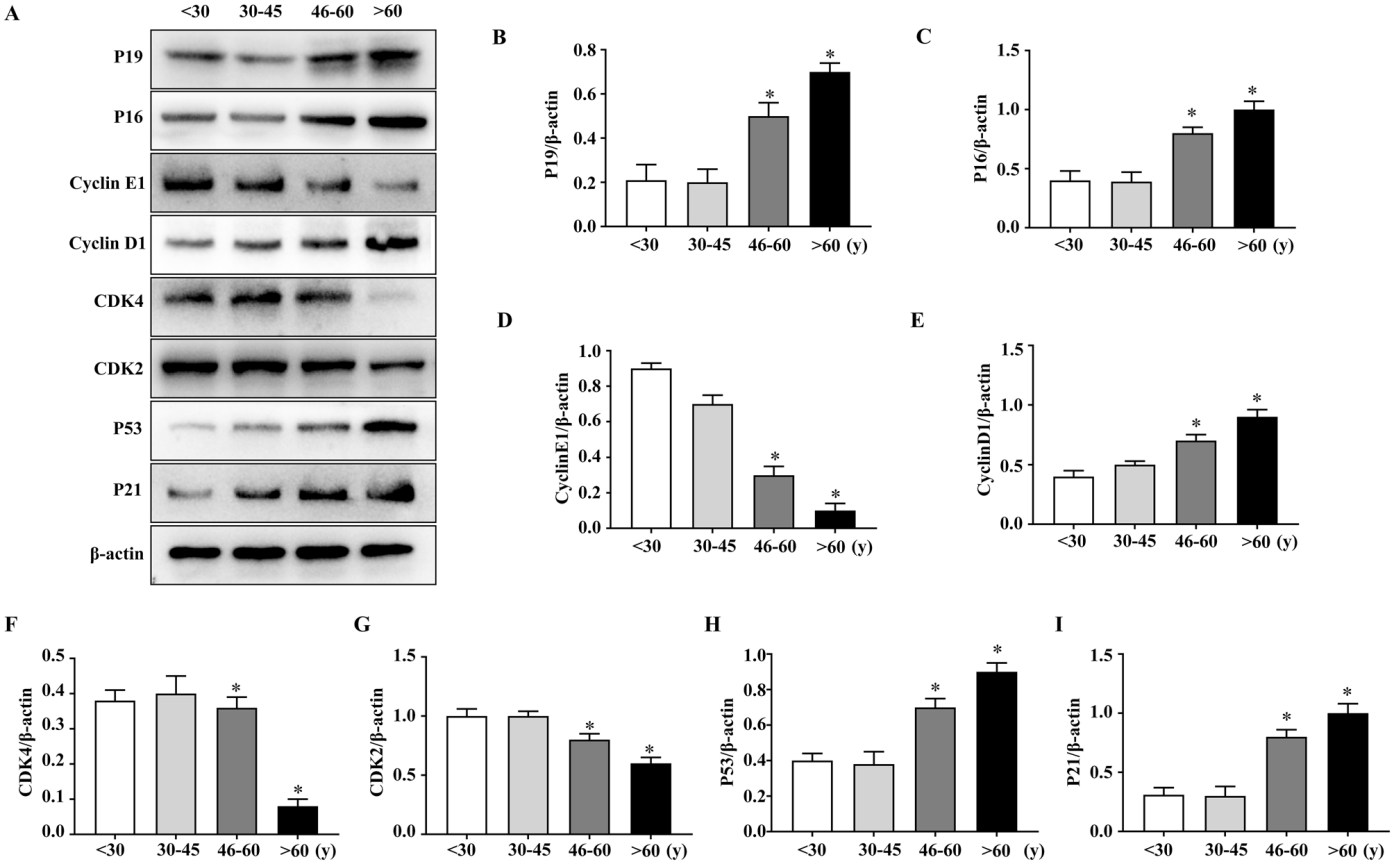
Differences in the expression levels of several key regulatory proteins in hBMHCs from different age groups. (**A**) P16, P53, P21, CyclinD1, P19, CDK2, CyclinE1 and CDK4 expression levels were detected by western blot analysis, full-length blots are presented in Supplementary Figure S1. (**B–I**) Graphical representation of western blot results in each sample group. *p < 0.05: when compared with the < 30 years group.

We measured the expression levels of aging-related proteins using total monocytes after the removal of adherent cells. Notably increased expression of P16, P19, P21 and P53 proteins was observed in the 46–60 and > 60 years age groups compared to that in the < 30 years age group, but there was no marked difference in expression of these proteins between the 30–45 and < 30 years age groups (Fig. 5A-C,H,I). Expression of CDK2 and CyclinE1 proteins was significantly lower in the 30–45, 46–60 and > 60 years age groups compared to that in the < 30 years age group (Fig. 5A,G,D). CDK4 protein expression was also notably reduced in the > 60 years age group when compared with that in the < 30 years age group, whereas no marked difference was observed in any of the other comparisons (Fig. 5A,F). CyclinD1 protein expression significantly increased in the 30–45, 46–60 and > 60 years age groups compared to that in the < 30 years group (Fig. 5A,E).

## Discussion

Stem cells are known for their capacity for self-renewal, damaged tissue repair and multidirectional differentiation. Previous studies^9–11^ have demonstrated a progressive decline in stem cell function and dysregulated stem cell proliferation and differentiation with increasing age. HSCs first proliferate and differentiate into hematopoietic progenitor cells under the regulation of the bone marrow-induced hematopoietic microenvironment^12^, and the latter further proliferate and differentiate into various peripheral blood cell types^13^. However, increasing age activates the senescence signaling pathways, thereby gradually decreasing the number of HSCs^14^, eventually leading to the exhaustion of the HSC pool and a decline in their function. The mechanism underlying hematopoietic stem cell senescence primarily involves DNA damage^15^, telomere shortening^16^, elevated levels of oxidative stress^17^, metabolic changes, and changes in the hematopoietic microenvironment^18^. Previous studies^7^ have explored the temporal dynamics of hematopoietic stem cell senescence in mice, laying a foundation for research on the dynamic biological patterns of human hematopoietic stem cell senescence. However, studying hematopoietic stem cell senescence in humans is far more difficult and complicated than in mice. BMHCs are derived from HSCs, and the properties of senescent BMHCs can reflect the level of hematopoietic stem cell senescence. Therefore, we used these cells to evaluate the regulatory mechanisms responsible for human hematopoietic cell senescence to provide theoretical and technical basis for future research into the temporal dynamics of hematopoietic stem cell senescence.

A decrease in cell proliferation and differentiation capacity is an important manifestation of cellular senescence^19^. Previous studies^19^ suggest that senescent HSCs experience a significant decrease in their proliferation capacity, which is consistent with our findings. We also found that senescent hematopoietic cells experienced G1-phase cell cycle arrest, supporting our hypothesis of cell growth arrest^20^ and reduced cellular proliferation in response to cellular senescence, which is similar to previous research findings. Differentiation capacity is another important indicator of stem cell function, and mixed hematopoietic progenitor cells or mixed colony forming units (CFU-Mix) are the earliest myeloid hematopoietic progenitor cells derived from HSCs, with colonies comprising erythrocyte progenitors, granulocytes, and megakaryocytes. The number of CFU-Mix derived from an equal number of BMHCs and the number of cells within each colony reflect the multidirectional differentiation capacity of the HSCs^21,22^. Here, we found that an increase in the age of the BMHC donor was accompanied by a gradual decrease in the number of CFU-Mix derived from each hBMHC and a reduction in the total number of cells in each of the colonies. At the same time, among our samples collected from clinical outpatients, we found that in the group aged 30–45 years, the proportion of women was higher than that of men, while the proportion of men was much higher than that of women in the donor group over 45 years old. Whether this phenomenon suggests that the relationship between gender and age in hematological outpatients is related to the types of diseases and the proportion of diseases in the clinic needs to be studied further, preferably with a larger sample size.

Oxidative stress plays an important role in the induction and maintenance of senescence and in the damage that senescent cells can cause to the body^23–26^. This theory claims that senescence can cause oxidative damage following an accumulation of ROS (the byproducts of normal metabolism in the body), which represses gene expression by damaging the cell’s DNA structure, impairing basic cellular functions and structure, thereby initiating a negative feedback loop directly linked to the aging process. MDA represents a class of lipids formed by the reaction of unsaturated fatty acids and oxygen free radicals on the surface of cellular membranes and can reflect the level of lipid oxidation in the body^27^. Thus, MDA is an important indicator of the extent of oxidative cellular damage. We compared ROS concentration and intracellular MDA expression in hBMHCs obtained from individuals of different age groups. Our observations revealed that both ROS and MDA levels gradually increase with age, and these effects are most prominent in the > 60 years group. Natural antioxidant systems include superoxide dismutase (SOD)^28^, glutathione (GSH)^29^, and various other enzymes. Our observations revealed that both SOD and GSH activities decrease with increasing age.

Studies suggested that the p53-p21 pathway is activated during HSC senescence in mice^30,31^ and that the p19 protein inhibits p53 degradation during the cell cycle, thereby increasing its expression in cells and activating its downstream target p21. In turn, p21 specifically inhibits the production CyclinE1-CDK2 complexes and inhibits CDK2 protease activity resulting in cell cycle arrest^32^. Meanwhile, p16 suppresses CDK4 protease activity by inhibiting its binding of CyclinD1 during the cell cycle, which eventually inhibits the expression of genes associated with S-phase and induces G1-phase cell cycle arrest^32,33^. These observations are consistent with the findings of our study, which suggest that CDK2 and CDK4 protease activities are necessary for the G1 to S-phase transition, and that CyclinE1 and CyclinD1 are important regulatory subunits of CDK2 and CDK4, respectively in hBMHCs. Our data revealed that the expression of CDK2, CDK4 and CyclinE1 gradually decreases with age, which results in an increased expression of p16 and p19 encoding genes and thus, contributes to the inhibition of cyclin transcription. Collectively, this study identified an increase in senescent hBMHCs with age, which are characterized by cell cycle arrest, high oxidative stress levels, and the activation of proteins related to aging.

## Conclusions

Aging is accompanied by gradual senile changes in hBMHCs including reduced cellular proliferative capacity. The possible reasons for this phenomenon include the activation of senescence-associated genes, expression of senescence-associated proteins, activation of senescence-related signaling pathways p16, p53-p21, increased oxidative stress, and damage caused by an imbalance between the generation of free radicals and the activity of the antioxidant system. These findings shed new light on senescence and open novel avenues for future research studies aimed at reversing aging and lowering the incidence of age-related diseases via drug intervention. Given the importance of these findings, we believe that this topic will continue to be a topic of interest for further investigation.

## Supplementary Information

Supplementary Figure S1.

## Data Availability

All data generated or analyzed during this study are included in this published article [and its supplementary information files].

## Abbreviations

CFU: Colony forming unit
GSH: Glutathione
SOD: Superoxide dismutase
DMEM: Dulbecco’s modified eagle medium
FBS: Fetal bovine serum
hBMHC: Human bone marrow hematopoietic cells
HSCs: Hematopoietic stem cells
MDA: Malondialdehyde
ROS: Reactive oxygen species
SA-β-gal: Senescence-associated-β-galactosidase
