# Supplementary figures and images for: Dynamic biological characteristics of human bone marrow hematopoietic stem cell senescence
Source: Sci Rep. 2022 Oct 12;12:17071. doi: 10.1038/s41598-022-21387-x (PMC9556752; doi:10.1038/s41598-022-21387-x)

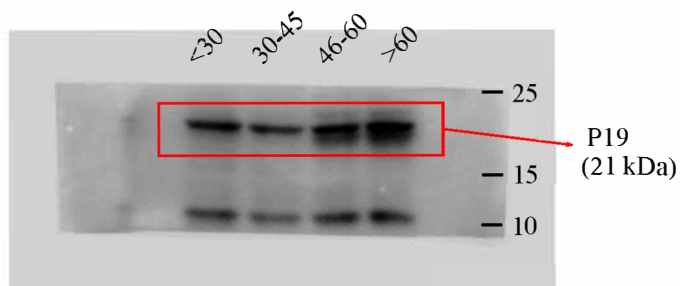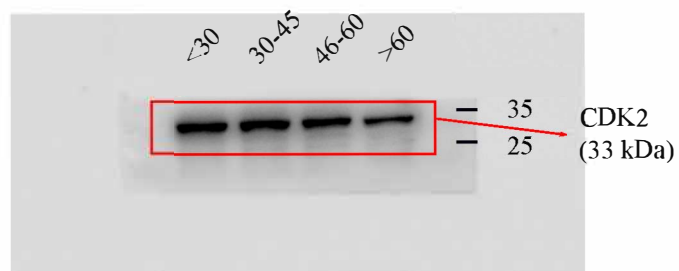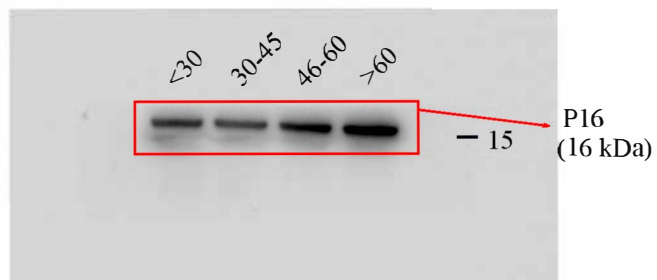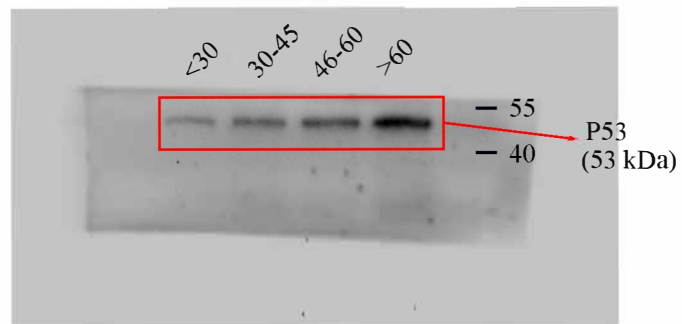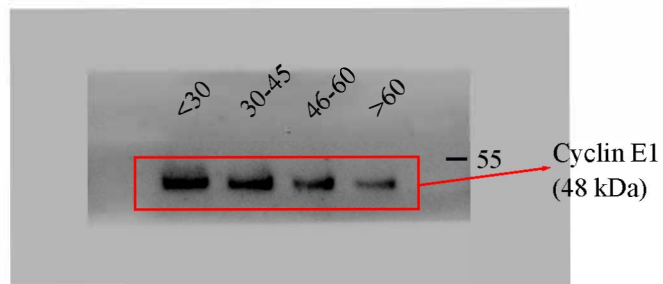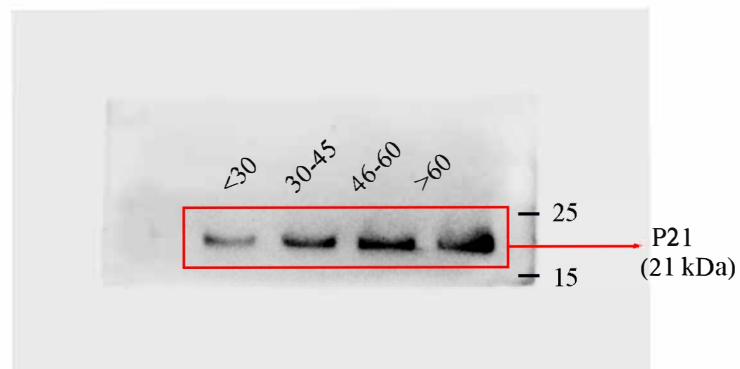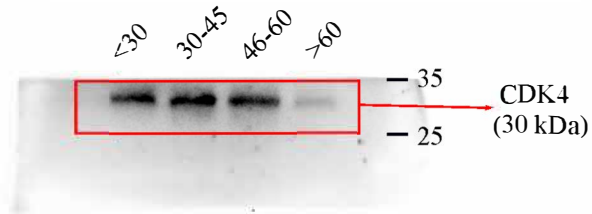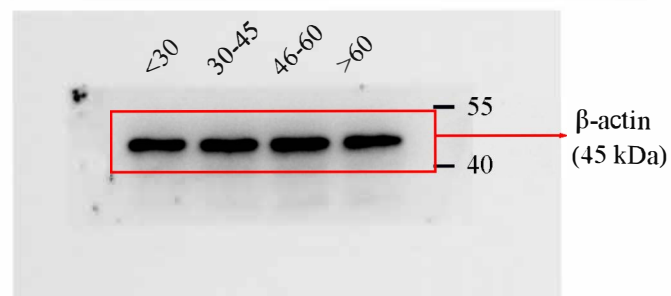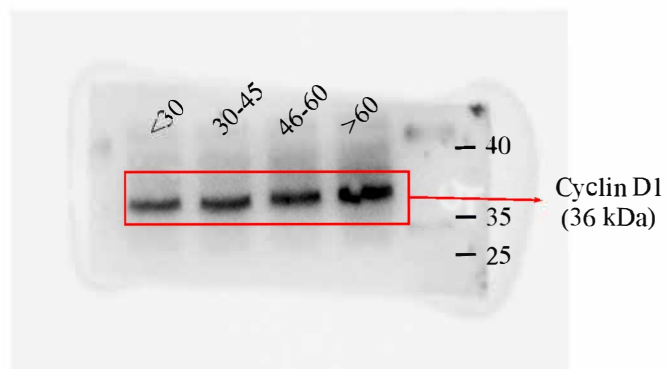

Supplement: Supplementary file 1 — Supplementary Figure S1. [file 41598_2022_21387_MOESM1_ESM.pdf]
